# A commentary on ‘Local excision as a viable alternative to hysterectomy for early-stage cervical cancer in women of reproductive age: a population-based cohort study’

**DOI:** 10.1097/JS9.0000000000001218

**Published:** 2024-02-21

**Authors:** Jianxia Zhang, Huiling Qiu, Yinxia Qing, Yanxia Hu, Huaxiang Deng

**Affiliations:** Department of Gynaecology, Boao Yiling Care Center, Qionghai, 571400 Hainan, People’s Republic of China

Cervical cancer is one of the primary causes of cancer-related fatalities in women. It ranks fourth in the world among women’s malignant tumor incidence, with 85% of cases occurring in developing nations^1^. Chen *et al*.^2^ conducted a retrospective population analysis on female patients diagnosed with primary cervical cancer for the first time. It appears that hysterectomy is still the best course of treatment for patients who do not require reproductive assistance, and fertility-sparing surgery (FSS) through local excision is a feasible option that can help patients under 40 with stage IA cervical cancer achieve a well-balanced outcome between tumor control and fertility preservation.

Data from the Surveillance, Epidemiology, and End Results (SEER) Program were used in this retrospective cohort analysis, which comprised 18 519 patients with cervical cancer who were of reproductive age and 2268 fatalities that were noted. Seventy-one percent of patients had a hysterectomy and 17.0% had FSS by local excision. The results of the study showed that while overall survival (OS) and disease-specific survival (DSS) of local excision were similar to those of hysterectomy in patients under 39, they were much worse in patients over 40 than they were with hysterectomy. Furthermore, in patients with stage IA cervical cancer, the OS and DSS of local excision were comparable to hysterectomy; however, in patients with stage IB cervical cancer, the OS and DSS of local excision were less favorable than hysterectomy.

This essay has several benefits and sources of inspiration. The trial’s high sample size and retrospective population study design made it possible to detect any clinically meaningful group differences, which was one of its benefits. As a result, the study provides trustworthy findings to direct care. Still, there were a lot of limitations. First, only American patients were included in this study. Patients’ management approaches may differ significantly from those in other nations. When extrapolating the findings to patients worldwide, these distinctions must be taken into consideration. Second, the research design’s primary flaw – along with all the well-known associated biases – is that it is nonprospective^3^. Finally, there are still a lot of confounding variables among women who have fertility-sparing surgery because it is unclear how many of these women were actively trying to conceive or had access to reproductive care. This is one of the study’s main drawbacks^4^.

This study explores if FSS via local excision could result in a comparable or even better prognosis than hysterectomy, as well as the present applications of local excision for early-stage cervical cancer. Large-scale, retrospective cohort research establishes the groundwork for future, more extensive trials by showing that patients who undergo local excision can attain a well-balanced result between tumor management and fertility preservation. This innovative strategy offers insightful direction and motivation for the ongoing advancement of this field of study. The goal of recent advancements in the surgical management of cervical cancer in its early stages has been to reduce morbidity without sacrificing efficacy^5^. To verify this result, more thorough and multicenter research is needed.

## Ethical approval

This manuscript is a comment. Don’t need ethical approval.

## Consent

This manuscript is a comment. Don’t need patient consent.

## Sources of funding

Not applicable.

## Author contribution

J.Z. and H.Q.: study concept or design, data collection, data analysis or interpretation, and writing the paper; Y.Q. and Y.H.: data analysis or interpretation; H.D.: study concept or design, writing, and revising the paper.

## Conflicts of interest disclosure

This manuscript is a comment without conflicts of interest.

## Research registration unique identifying number (UIN)

This manuscript is a comment. Don’t need UIN.

## Guarantor

Huaxiang Deng.

## Data availability statement

This manuscript is a comment. Don’t need a Data availability statement. However, all the data from the current study are publicly available.

## Provenance and peer review

This manuscript is a comment without being invited.
